# The obesity-brain axis: a comprehensive review of neurological complications and therapeutic interventions in metabolic syndrome

**DOI:** 10.1186/s13098-026-02115-1

**Published:** 2026-03-07

**Authors:** Amin Roshdy Soliman, Ebtesam Fahmy, Rabab Mahmoud Ahmed

**Affiliations:** Kasr Alainy Faculty of medicine, Cairo University, El Saray Street Manial,El Manial, Cairo, 11562 -Manial Egypt

**Keywords:** Obesity, Metabolic syndrome, Neurological disorders, Cognitive impairment, Neuroprotection, Dietary intervention

## Abstract

**Background:**

Obesity has emerged as a major global health issue, affecting multiple organ systems. Within the central nervous system obesity causes a series of disruptions that can significantly affect neurological function. Identifying obesity as a modifiable risk factor presents opportunities for preventive and therapeutic strategies that may significantly diminish neurological sequelae.

**Objective:**

This narrative review aims to summarize current evidence on how obesity contributes to different neurological diseases and focusing on biological mechanisms linking obesity to these conditions, outlines the characteristic clinical presentations of obesity-related neurological diseases across different age groups and potential therapeutic strategies.

**Methods:**

This narrative review integrates findings from comprehensive search of PubMed, EMBASE, and Cochrane Library to investigate how obesity and metabolic syndrome relate to a broad spectrum of neurological disorders. After screening 1,950 records, 48 studies were included supplemented by nine manually identified articles.

**Results:**

Obesity triggers a range of biological changes in the nervous system such as increased oxidative stress, persistent low‑grade inflammation, disruption of the blood–brain barrier, and impaired mitochondrial function. Together, these changes raise the risk of several neurological problems, including cognitive decline, Alzheimer’s disease, stroke, faster progression of multiple sclerosis, greater epilepsy‑related complications, transformation of episodic into chronic migraine, idiopathic intracranial hypertension, and various peripheral neuropathies. The impact of body mass index on neurological health differs across diseases and age groups with obesity in midlife representing a high risk. Lifestyle‑based strategies especially Mediterranean or ketogenic dietary patterns, regular physical activity and weight reduction show encouraging potential in reducing these neurological risks.

**Conclusions:**

Obesity is a modifiable contributor to many neurological disorders. Identifying at‑risk individuals early and adopting healthier daily habits, following tailored diets and managing weight effectively may help lessen the neurological consequences of obesity. Continued research is essential to clarify underlying mechanisms and refine treatment strategies for different patient groups.

## Introduction

One of the most urgent public health issues of the twenty-first century is the obesity epidemic. Obesity is now widely acknowledged by physicians worldwide as a complex, multifactorial disease with a significant impact on human health, rather than just a cosmetic problem. The landscape of managing chronic diseases has changed significantly as the prevalence of obesity continues to rise sharply in both developed and developing countries [1]. With values of 18.5–24.9 kg/m² considered normal, 25.0–29.9 kg/m² overweight, 30.0–39.9 kg/m² obese, and ≥ 40.0 kg/m² severely obese, body mass index is still the most commonly used metric for categorising body composition [2, 3].

The World Health Organization defines obesity as excessive or abnormal body fat accumulation. The aetiology of obesity is complex involving genetic predisposition, behavioural, societal and environmental factors. While genetics may make certain populations more prone to weight gain, environmental and behavioural factors predominate in most cases. Overconsumption of energy-dense foods and refined carbohydrates in conjunction with reduced physical activity and sedentary lifestyles is considered the main cause of weight gain in most cases. Endocrine abnormalities like hyperinsulinism, hypercortisolism, ovarian dysfunction, and hypothyroidism have also been associated with obesity development. Certain drugs such as steroid hormones and psychoactive agents can also induce weight gain [4, 5].

**Fig. 1:**
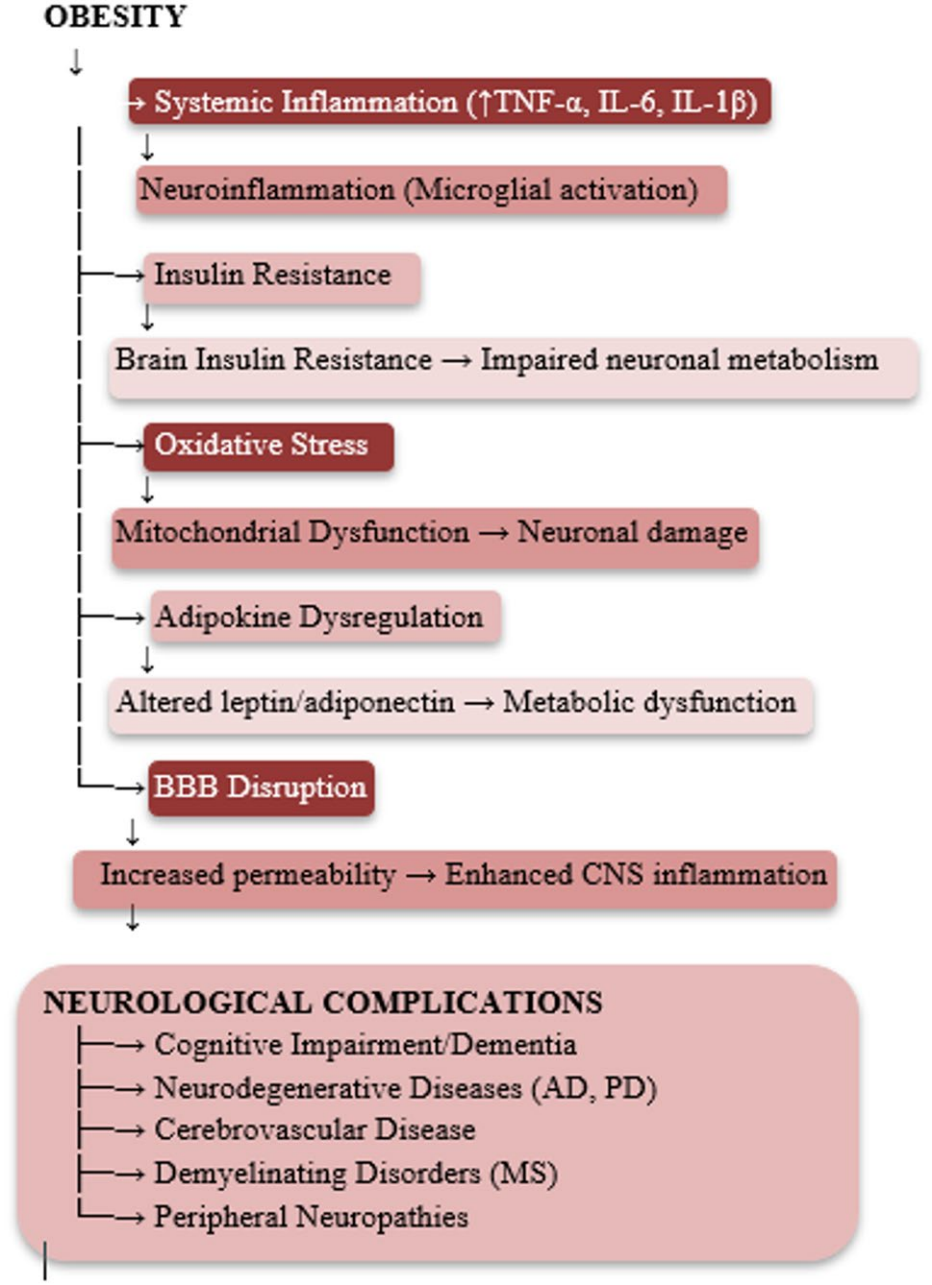
Pathophysiological Mechanisms Linking Obesity to Neurological Dysfunction. TNF-α = Tumor Necrosis Factor-alpha, IL-6 = Interleukin-6, IL-1β = Interleukin-1 beta, BBB= Blood-Brain Barrier, CNS= Central Nervous System, AD= Alzheimer’s Disease, PD=Parkinson’s Disease, MS=Multiple Sclerosis

Within the central nervous system, obesity causes a series of disruptions that can significantly affect neurological function (Fig. 1). These include more oxidative stress, chronic inflammation, abnormal protein accumulation, mitochondrial dysfunction, hormonal imbalances, insulin resistance, and blood-brain barrier impairment [6]. Together, these processes interfere with synaptic plasticity and neurogenesis leading to neuronal injury contribute to cognitive decline [7]. Obesity that develops during early life can produce long-lasting alterations in the innate immune system even after metabolic measures return to normal [8].

This study offers a comprehensive review of neurological conditions linked to obesity. Our work covering cognitive, neurodegenerative, demyelinating, cerebrovascular, epileptic, and peripheral nervous system diseases whereas previous reviews have usually concentrated on single neurological conditions or specific mechanistic pathways. We uniquely integrate potential underlying mechanisms with clinical conditions and applicable management methods supported by comparative summary tables and figures. In order to improve clinical practice, it lists the common clinical manifestations of neurological disorders associated with obesity in various age groups. This review offers a more expanded and practically useful viewpoint than previous reviews by addressing inconsistent findings, evidence gaps, and future research goals across several neurological domains. These contributions highlight the novelty and added value of our work.

### The methodology adopted for this narrative review regarding the selection of studies, was structured as follows

A focused search of the literature was carried out. The terms: Obesity, neurological complications, Alzheimer’s disease, multiple sclerosis, stroke, epilepsy, migraine, idiopathic intracranial hypertension, peripheral nervous system diseases, cognitive impairment, dementia, neurodegeneration, demyelinating disease, cerebrovascular disease, seizures, idiopathic intracranial hypertension (IIH), neuropathies, body mass index, adult, paediatric, child, and adolescent were all included in the literature search of the PubMed, EMBASE, and Cochrane Library databases from the beginning to the present. It includes studies that address the epidemiology, mechanisms, clinical features, risk factors, or therapeutic interventions associated with obesity-related neurological disorders across all age groups. We included original articles, systematic reviews, meta-analyses, clinical guidelines, and case series. 1,950 articles are screened and 48 articles were included. In order to find more studies that were suitable to the review’s general scope, we also manually searched related articles (additional nine studies). Studies not in English, lacking sufficient methodological detail or not providing obesity-specific neurological data, conference abstracts were excluded during screening.

## Pathophysiological mechanisms linking obesity to neurological dysfunction

### Metabolic and inflammatory pathways

Obesity leads to widespread metabolic disturbances, one of the most important being a persistent low-grade inflammatory state often referred to as (meta-inflammation). In this setting, adipose tissue acts much like an endocrine organ, releasing pro-inflammatory cytokines such as tumour necrosis factor-alpha, interleukin-6, and interleukin-1β. These molecules can cross the blood–brain barrier, and activate microglia and astrocytes, setting off and sustaining inflammatory processes within the brain [9].

### Insulin resistance and central nervous system function

Peripheral insulin resistance, a hallmark of obesity and metabolic syndrome, extends to the central nervous system. Brain insulin resistance impairs neuronal glucose metabolism, synaptic plasticity, and neuroprotective signalling pathways. This dysfunction particularly affects hippocampal regions critical for memory formation and consolidation [10].

### Oxidative stress and mitochondrial dysfunction

obesity correlates with increased reactive oxygen species production and diminished antioxidant capacity. Mitochondrial impairment in neurons affect energy production and cellular homeostasis making cells vulnerable to apoptotic signalling and accelerated aging [11].

### Blood-Brain barrier integrity

Obesity-associated inflammation and metabolic dysregulation affect blood-brain barrier integrity facilitating entry of peripheral inflammatory mediators, toxins, and immune cells into the central nervous system. This breach raises neuroinflammatory responses and accelerates neurodegenerative processes [12].

### Epigenetic modifications

Recent evidence showed that obesity particularly during early life induces lasting epigenetic changes in innate immune cells which persist beyond resolution of metabolic abnormalities, suggesting that obesity’s neurological effects may extend far beyond periods of active weight excess [13].

## Neurological comorbidities associated with obesity

### Cognitive impairment and dementia

**Fig. 2:**
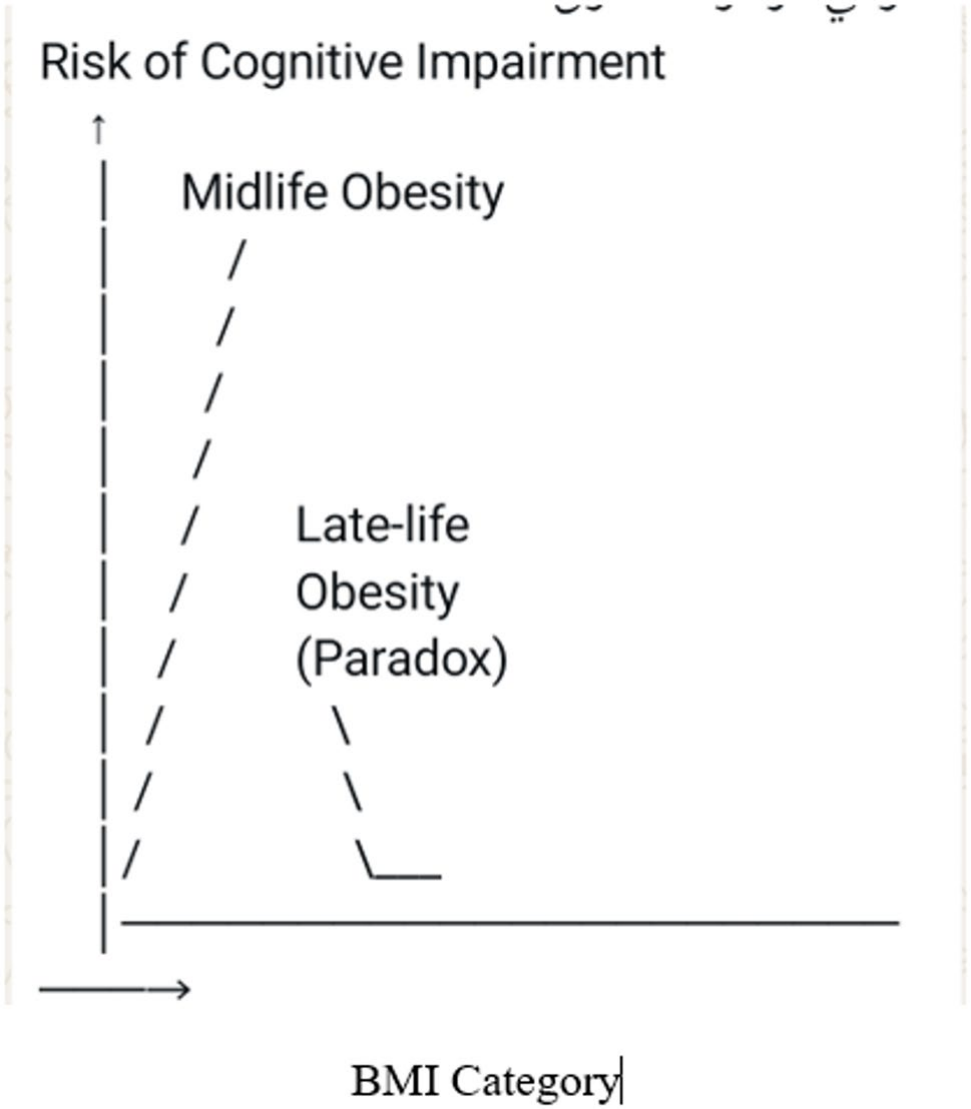
Age-Dependent Relationship Between body mass index (BMI) and Cognitive Outcomes

The relationship between obesity and cognitive function showed complex age-dependent patterns (Fig. 2). In young and middle-aged adults’ obesity correlates positively with cognitive impairment affecting executive function, processing speed, and memory domains [14]. However, epidemiological studies reveal that older adults with obesity may demonstrate reduced frequency of cognitive impairment a phenomenon termed the (obesity paradox) [15].

Multiple mechanisms contribute to obesity-related cognitive decline including cerebrovascular insufficiency, neuroinflammation, insulin resistance, and increased brain atrophy. The timing of obesity appears critical with midlife adiposity showing greater risk than late-life obesity for subsequent cognitive deterioration.

### Alzheimer’s disease

strong evidence links obesity with increased Alzheimer’s disease risk and earlier disease onset. High body mass index during midlife appears to form an important component in underlying Alzheimer’s pathophysiology and accelerated cognitive decline [16]. Possible mechanisms include enhanced amyloid-beta deposition, tau phosphorylation, synaptic dysfunction, and hippocampal atrophy.

Paradoxically the relationship between body mass index and Alzheimer’s disease reverses in later life, with some researches suggesting that higher body mass index may decreases disease burden or aggressiveness in established Alzheimer’s disease [17]. The apparent paradox that obesity raises the risk of dementia in midlife and appears to be protective in later life may be the result of both methodological bias and biological mechanisms. Since early Alzheimer’s pathology often causes metabolic changes and unintended weight loss years before clinical diagnosis, reverse causation is important. As a result, low BMI in old age is not a protective state but rather a sign of developing disease. Results may be affected by survival bias because obese older adults may be exceptionally resilient. Furthermore, studies that account for survival or health issues associated with obesity may be subject to collider bias [18]. The inaccuracy of BMI in older adults with sarcopenia and altered fat distribution further complicates interpretation. Numerous biological theories as metabolic reserve, leptin-related neurotrophic effects, inflammatory preconditioning, and vascular factors that may aid in maintaining cerebral perfusion, but this doesn’t mean that obesity is helpful.

Clinically, body composition is more informative than BMI, late-life weight loss should be handled cautiously, and midlife is still the crucial window for preventing obesity-related dementia. Repeated weight measurements, prolonged follow-up, body composition measurements, genetic stratification, and modelling of weight patterns throughout ageing should all be included in future studies [19].

### Parkinson’s disease

The association between body weight and Parkinson’s disease remains controversial, with conflicting evidence across studies. While some studies find no significant relationship between body mass index and future Parkinson’s disease development; others identify overweight status as a potential risk factor compared with normal body mass index [20, 21].

Underweight individuals showed elevated Parkinson’s disease risk, with this effect augmented in patients with associated diabetes mellitus [22]. These findings suggest complex non-linear relationships between metabolic status and Parkinson’s disease pathogenesis that need further investigation.

### Multiple sclerosis

Obesity prevalence is significantly elevated among multiple sclerosis patients especially females [23]. Body mass index correlates significantly with disease disability as measured by Expanded Disability Status Scale scores [24]. Higher body mass index is linked to a higher progression from clinically isolated syndrome to multiple sclerosis, increased relapse frequency, accelerated disability progression, and more pronounced cognitive deterioration and brain atrophy [25].

Evidence suggests obesity increases multiple sclerosis susceptibility through interactions with genetic and environmental risk factors. Obese patients show inferior responses to disease-modifying therapies compared with normal weight individuals. The relationship between increasing body mass index and diminished treatment response underscores the importance of weight management in multiple sclerosis care [26].

Paediatric-onset multiple sclerosis also demonstrates strong associations with obesity, particularly in girls, with odds ratios of 1.58, 1.78, and 3.76 for overweight, moderate obesity, and extreme obesity, respectively [27].

### Cerebrovascular disease

Obesity considered one of the most significant modifiable risk factors for ischemic stroke, with prevalence ranging from 6 to 20% across European countries and 34% in the United States [28]. Debate continues regarding whether obesity itself constitutes an independent stroke risk factor or whether metabolic syndrome components mediate this relationship.

Epidemiological cohorts and meta-analyses indicate that obesity confers a 7–37% higher risk of stroke compared with normal-weight individuals corresponding to roughly a 4% increase in risk for each 5 kg/m² rise in body mass index, independent of metabolic syndrome factors. Obese individuals experience stroke at younger ages and demonstrate poorer outcomes with increased complications following cerebrovascular events [29].

### Epilepsy

Overweight and obesity prevalence is elevated among epilepsy patients [30]. Several antiepileptic agents such as valproic acid and pregabalin are associated with weight gain and adverse metabolic effects [31]. Obesity adversely affects medication compliance and seizure control, with patient body mass index potentially predicting antiepileptic drug response [32].

### Migraine

Chronic migraine risk increases progressively from normal weight through overweight to obese categories. Individuals with migraine at healthy weight face approximately 3% annual risk of chronic headache development. This risk triples with overweight status and quintuples with obesity. Obesity is associated with a higher likelihood of progression from an episodic to a chronic headache pattern, functioning as a risk amplifier rather than direct migraine cause [33].

### Idiopathic intracranial hypertension

Obesity represents a major risk factor for idiopathic intracranial hypertension, with the association between elevated body mass index and this condition widely recognized. Weight loss is considered the only definitive cure. Higher body mass index is associated with a greater tendency toward more severe papilledema and accompanying visual impairment necessitating close monitoring of patients with very high BMI for visual field progression [34].

### Peripheral neuropathies

Prospective studies have identified associations between higher body mass index, greater waist circumference, and an increased risk of Guillain–Barré syndrome [35]. Similarly, elevated body mass index has been linked to a higher incidence of Bell’s palsy [36] especially in pediatric populations with obese individuals exhibiting slower recovery trajectories.

Relationships exist between elevated body mass index and carpal tunnel syndrome as well as meralgia paresthetica. Slender individuals (body mass index < 20) demonstrate 16% carpal tunnel syndrome incidence compared with 39% in obese patients (mean body mass index > 29). Obese individuals face 2.5-fold higher carpal tunnel syndrome probability compared with slender individuals [37].

Table 1 summarizes BMI categories together with their associated neurological risk levels, offering a practical framework for understanding how weight may influence vulnerability. Table 2 provides an integrated overview of neurological disorders linked to obesity, highlighting both the strength of these associations and the age-related patterns that shape how these conditions emerge across the lifespan.

**Table 1:** BMI classification and neurological risk categories

BMI Category	BMI Range (kg/m²)	Classification	General Neurological Risk
**Underweight**	< 18.5	Underweight	Increased (PD, stroke)
**Normal**	18.5–24.9	Normal weight	Baseline
**Overweight**	25.0–29.9.0.9	Overweight	Moderately increased
**Class I Obesity**	30.0–34.9.0.9	Obese	Significantly increased
**Class II Obesity**	35.0–39.9.0.9	Obese	Markedly increased
**Class III Obesity**	≥ 40.0	Severely obese	Severely increased

**Table 2:** Summary of Obesity-Associated Neurological Disorders

Neurological Condition	Strength of Association	Key Findings	Age Related Patterns
**Cognitive Impairment**	Strong	Positive correlation in young/middle-aged adults	Paradoxical in elderly
**Alzheimer’s Disease**	Strong	Midlife BMI increases risk	Late-life high BMI may be protective
**Parkinson’s Disease**	Weak/Conflicting	Inconsistent findings; underweight increases risk	Variable
**Multiple Sclerosis**	Strong	Higher relapse rates, disability progression, reduced treatment response	Consistent across ages
**Ischemic Stroke**	Strong	7–37% increased risk; younger age at onset	Consistent across ages
**Epilepsy**	Moderate	Affects drug response and seizure control	Consistent across ages
**Chronic Migraine**	Strong	5-fold increased risk with obesity	Consistent across ages
**IIH**	Very Strong	Major risk factor; weight loss is curative	Consistent across ages
**Peripheral Neuropathies**	Moderate to Strong	CTS, Bell’s palsy, GBS, meralgia paresthetica	Consistent across ages

## Therapeutic interventions and management strategies

### Weight reduction and caloric restriction

Many of harmful neurological effects of obesity are reversible through weight loss. Caloric restriction proves more effective than exercise alone for initial weight reduction. In humans, fasting prevents and treats metabolic syndrome, a major risk factor for many neurological diseases. Fasting improves cognition, attenuates age-related cognitive decline, slows neurodegeneration, lessen brain injury after stroke, support better functional recovery, and ease both the pathological and clinical manifestations of epilepsy and multiple sclerosis [38].

### Surgical interventions

patients with very high BMI and idiopathic intracranial hypertension require close monitoring for visual field loss progression with early consideration for definitive surgical intervention especially in patients with multiple risk factors including black race, male sex, anemia, hypertension, and morbid obesity [39]. As the metabolic surgery represents a highly invasive therapeutic option, its use should be reserved for situations in which dietary and lifestyle interventions as well as new pharmacologic therapies have been attempted or are contraindicated [40].

Bariatric surgery showed significant benefits for migraine management. A recent meta-analysis that pooled six studies involving 1,899 patients who underwent sleeve gastrectomy or gastric bypass showed significant improvement in migraine outcomes, a significant reduction in monthly migraine days alongside decrease in migraine severity and the frequency of disabling attacks [41].

### Dietary interventions

***Mediterranean Diet***: dietary and physical activity interventions can influence both obesity and the course of neurological diseases. The Mediterranean diet stands out as one of the most effective for lowering the risk of neurodegenerative conditions. In multiple sclerosis, dietary changes have been shown to reduce fatigue, improve disability scores, and lessen symptoms of depression. People who follow a Mediterranean diet al.so tend to report better quality of life, lower disability levels, reduced fatigue, and a lower likelihood of developing multiple sclerosis. In Alzheimer’s disease, dietary interventions can enhance daily functioning and overall quality of life. Strong adherence to the Mediterranean diet is consistently linked to a reduced risk of developing Alzheimer’s disease [42].

In Parkinson’s disease, Mediterranean diet produces significant cognitive improvements of executive function, language, attention, concentration, and active memory. The MIND diet (Mediterranean-DASH Intervention for Neurodegenerative Delay) associates with reduced parkinsonism risk and slower progression in older adults [43].

***Ketogenic Diet***: Beyond established epilepsy applications and uses for non-neurological conditions including heart disease, diabetes, obesity, autism, glioblastoma, and certain cancers, recent studies demonstrate promising results for neurodegenerative diseases [40, 44]. However, most published studies in neurodegenerative diseases report short-term interventions where adverse cardiometabolic effects are less pronounced than in long-term lifestyle use and this needs larger, longer and better-controlled trials before drawing definitive conclusions [40, 44].

### Physical activity

In multiple sclerosis exercise helps in symptom management, functional recovery, quality of life enhancement and overall wellbeing promotion. For Alzheimer’s disease, physical activity temporarily enhances attention, executive functions, and language abilities. In Parkinson’s disease, moderate-intensity physical activity correlates with enhanced global cognition, visuospatial perception, memory, and executive function [45].

Therapeutic interventions and management strategies and disease-specific recommendations for obesity management are summarized in Tables 3 and 4..

**Table 3:** Therapeutic interventions for Obesity-Related neurological complications

Intervention Type	Specific Approach	Neurological Benefits	Level of Evidence
**Weight Loss**	Caloric restriction	Reverses multiple complications; improves cognition	High
**Dietary - Mediterranean diet**		Reduced neurodegeneration risk; improved cognition in AD, PD, MS	High
**Dietary - Ketogenic diet**		Seizure control; potential neuroprotection	Moderate-High
**Physical Activity**	Moderate-intensity exercise	Cognitive enhancement; symptom management	High
**Surgical**	Bariatric surgery	Migraine frequency reduction; IIH management	Moderate
**Fasting - Intermittent fasting**		Metabolic syndrome prevention; neuroprotection	Moderate

**Table 4:** Disease-Specific Recommendations for Obesity Management

Condition	Specific Recommendations	Monitoring Requirements	Expected Outcomes
**Multiple Sclerosis**	Mediterranean diet; exercise; weight optimization before DMT initiation	BMI, disability scores, relapse rates	Reduced relapse rates; improved DMT response
**Idiopathic Intracranial Hypertension**	Aggressive weight loss; close monitoring of vision	Visual fields, papilledema grade, BMI	Prevention of vision loss
**Chronic Migraine**	Weight reduction; consider bariatric surgery if severe	Headache frequency, intensity, medication use	Reduced headache frequency and severity
**Stroke Prevention**	Weight loss; metabolic syndrome management	BMI, blood pressure, lipids, glucose	Reduced stroke risk
**Epilepsy**	Weight monitoring with AEDs; ketogenic diet consideration	Seizure frequency, drug levels, BMI	Improved seizure control

#### Clinical considerations across the lifespan

The relationship between obesity and neurological health varies substantially across different life stages, necessitating age-specific approaches to risk assessment, prevention, and intervention.

#### Pediatric obesity:

carries special neurological implications because it happens during critical periods of brain maturation. Metabolic dysregulation can disrupt prefrontal cortex development, myelination and synaptic pruning with possible lifelong effects [46]. Early-life obesity also causes lasting epigenetic changes and leads to prolonged metabolic stress which may explain stronger neurological disease associations in those with childhood-onset obesity [47]. One of the strongest examples is pediatric-onset multiple sclerosis, where obesity markedly increases risk especially in girls with odds ratios rising from 1.58 in overweight children to 3.76 in severe obesity [48].

Clinically, management requires regular BMI monitoring using age and sex-specific percentiles, assessment of executive function and academic performance, family-centered lifestyle interventions, attention to social determinants, psychological support, age-appropriate goals focused on healthy growth and vigilance for symptoms such as headaches, cognitive difficulties or other neurological signs. Prevention strategies include healthy nutrition from infancy, regular physical activity and outdoor play, reduced screen time, school-based wellness initiatives, and parental education to reinforce healthy behaviors.

#### Young and Middle-Aged adults:

Midlife represents a critical window for obesity’s neurological effects and potentially the most impactful period for intervention. Midlife is the period when obesity most strongly affect neurological health with clear evidence that excess weight between ages 40 and 65 increases the risk of dementia, cognitive decline and stroke making it the prime target for prevention [49].Weight gain in these years is shaped by occupational stress, sedentary routines, and irregular schedules, while reproductive factors such as pregnancy-related changes, PCOS, and menopause further affect risk especially in women [50].

Clinical care should focus on intensive management of metabolic and vascular risk factors, early cognitive screening in those with obesity or metabolic syndrome. healthy dietary patterns, regular physical activity, stress management, and treating sleep apnea remain essential. Even modest weight loss of 5–10% can provide metabolic improvement highlighting the value of sustainable long-term habits rather than rapid weight reduction.

#### Elderly populations:

Obesity management in older adults requires balancing benefits and risks, as late-life BMI is influenced by factors like the obesity paradox, by frailty and sarcopenia which make body composition more informative than BMI alone. Multimorbidity, mobility limits, cognitive decline, and social isolation all affect both obesity related risk and the feasibility of weight loss [18, 19].

Weight reduction is appropriate for functionally adequate elderly with recent weight gain or obesity-related complications, while weight maintenance is preferred in those with stable weight, advanced age, frailty, or recent illness. Weight loss is generally inappropriate in malignancy, cachexia, advanced dementia, or unintentional weight loss. For suitable candidates, modest goals, adequate protein intake, resistance training, gradual caloric reduction, geriatric assessment, medication review and close monitoring are essential with nutritional adequacy prioritized over aggressive dieting [51].

## Algorithm for weight management is provided below

### Clinical considerations across special populations

#### Pregnancy and postpartum:

Excessive gestational weight gain and postpartum weight retention increase long term neurological risk. Preconception counselling for obese women should address neurological implications and postpartum weight management represents an important preventive opportunity [52].

#### Individuals with established neurological disease:

In multiple sclerosis, optimizing weight before starting disease-modifying therapies may enhance treatment response [25]; in epilepsy, regular monitoring of weight is important because many antiepileptic drugs influence body weight [53] and a ketogenic diet may be considered for refractory seizures [54]; and among stroke survivors, weight control supports secondary prevention though interventions must account for post-stroke depression, functional limitations and reduced mobility [55].

#### Ethnic and Racial disparities:

Obesity prevalence and neurological disease risk vary across ethnic and racial groups. Culturally tailored interventions, addressing structural barriers to healthy lifestyles and recognition of differential genetic susceptibility are essential for equitable care [56, 57].


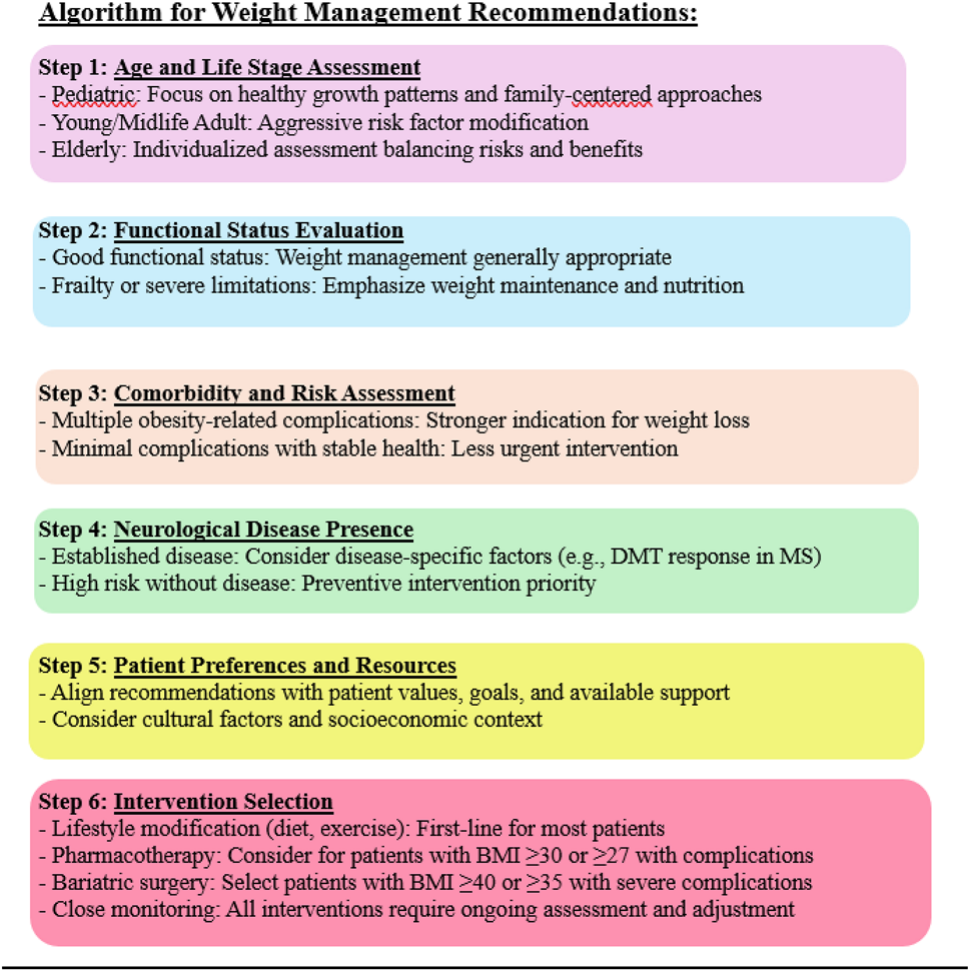


## Clinical implications and future directions

The reviewed evidence showed that obesity is an important modifiable risk factor for many neurological diseases. The relationships between obesity and neurological diseases were affected by different conditions and age groups requires individualized clinical care.

Healthcare providers should implement early screening for obesity-related neurological complications particularly in early and midlife when interventions may prove to be effective. Weight reduction strategies should be integrated into comprehensive neurological care protocols with recognition that pharmacokinetic considerations in obese patients may necessitate treatment modifications.

A thorough evaluation of study design, causality, and bias is necessary to fully understand the connections between obesity and neurological disorders. The majority of the evidence is observational which restricts the ability to make conclusions about causality. Although many documented associations satisfy some of the Bradford Hill criteria, confounding and reverse causation continue to be significant obstacles. Interpretation is further complicated by significant variation in BMI estimation, population characteristics, follow-up, outcome definitions and adjustment techniques across studies. Results can be inaccurate or contradictory due to biases like collider stratification bias when conditioning on obesity-related comorbidities and survival bias in older cohorts. When all factors considered, these methodological problems point to the necessity of evaluating obesity-neurological disease associations with caution and paying attention to study design, bias, and biological plausibility [18, 19].

### Future research priorities include

Rigid epidemiological designs, such as long-term cohorts with repeated body composition measurements, diverse populations, and integrated neuroimaging and biomarker sub-studies, should be employed in future research. Making causality clear is a top priority. Extended follow-up, trajectory calculation of weight change, body composition metrics other than BMI and age-stratified analyses are all necessary to address the obesity paradox.

While creating personalised interventions based on genetic profiles, metabolic phenotypes, specific weight loss goals and pharmacogenomic considerations, parallel precision medicine initiatives should identify high risk subgroups.

Future studies should assess multimodal approaches that can reduce the neurological risk associated with obesity such as combined diet and exercise regimens, medication-assisted lifestyle modifications, weight loss combined with cognitive training and social engagement-enhanced activity programs. Testing anti-inflammatory and neuroprotective treatments, comparing dietary patterns, evaluating the neurological effects of metabolic surgery and figuring out the best time, weight-loss thresholds and the effects of gradual versus rapid loss, weight cycling and intermittent fasting are additional priorities. Determining molecular pathways and creating early-detection biomarkers such as blood-based markers and neuroimaging signatures of brain vulnerability linked to obesity should be the focus of ongoing efforts.

### Limitations and bias of the study

There are various restrictions on this narrative review. The selection of studies may reflect author judgement because it is not a systematic review. Reliance on published research increases the possibility of publication bias. Cross-sectional studies included limits conclusions about causality and many studies did not address important confounders. Studies can differ widely in terms of design, duration, and inclusion criteria which make its generalisability is limited.

## Conclusion

Obesity negatively impacts neurological health through a number of overlapping mechanisms. The rising obesity epidemic suggests rising rates of neurological morbidity. Giving priority to obesity as a modifiable risk factor, early identification and timely management of obesity offers significant chances to prevent and reduce neurological complications in those at risk. There is hope for reducing the neurological effects of obesity when dietary interventions are combined with exercise and weight loss. To address this important public health issue, ongoing research aimed at uncovering underlying mechanisms and improving specific actions will be essential.

## Data Availability

All data generated in this study are included in this published article.

## Abbreviations

AD: Alzheimer’s disease
BMI: Body mass index
CTS: Carpal tunnel syndrome
DM: Diabetes mellitus
DMT: Disease modifying therapy
IIH: Idiopathic intracranial hypertension
PD: Parkinson’s disease
MS: Multiple sclerosis
